# Knowledge of stop the bleed techniques- a national survey

**DOI:** 10.1186/s13104-023-06540-7

**Published:** 2023-10-09

**Authors:** Siddharth Sheth, Rohan K. Mangal, Thor S. Stead, Latha Ganti

**Affiliations:** 1 Boston University, Boston, MA USA; 2 University of Miami Miller School of Medicine, Miami, FL USA; 3 The Warren Alpert School of Medicine, Providence, RI USA; 4 University of Central Florida College of Medicine, Orlando, FL USA

**Keywords:** Hemorrhage control, Tourniquets, Bleeding, Resuscitation

## Abstract

**Objective:**

This article presents the dataset titled “Do you know how to Stop The Bleed®? [1]” The dataset contains the survey responses of 200 US persons aged 16 years and above regarding their knowledge of hemorrhage control, based on the American College of Surgeons Stop The Bleed® (STB) course [2].

**Results:**

Two hundred adults in the United States completed this web-based survey, which consisted of a quiz to assess STB knowledge. Factors that were not statistically correlated to STB knowledge retention included age, sex, race and education level up to college level. On the other hand, resuscitation coursework (p = 0.004) and income (p = 0.049) were important determinants of Stop the Bleed® knowledge. In particular, participants with CPR certification (p = 0.020) and/or a postgraduate degree (p = 0.015) scored higher than their counterparts in this sample cohort.

## Introduction

The objective of this research is to assess knowledge of Stop the Bleed techniques in a US population [1]. Stop The Bleed® is a course designed by the American College of Surgeons in partnership with the US department of Defense [2]. The premise for the course is that life threatening bleeding can be most effectively stopped in a timely manner buy the person in closest proximity to the victim, and that is often a layperson. Initial hemorrhage control techniques however can be learned relatively easily by laypersons, hence the campaign- “Stop The Bleed- Save A LIfe.”

## Survey methods

Two hundred participants from the United States completed a questionnaire that includes a Stop the Bleed® training quiz. All participants were required to be at least 16 years old. This survey was administered through a research platform based on organic sampling and random device engagement. Its built-in algorithms prevent users from completing the survey multiple times using different devices; this security measure ensures each response is from a unique individual. The platform features over 700 million global users, of which only U.S. residents were considered for the study. For univariate distributions weighting was used. Participants’ responses were analyzed using JMP Pro 15 for Mac OS.

The survey did not include a screening question. All respondents were required to be 16 years of age or older to meet the inclusion criteria. Most questions were multiple choice (allowing one answer selection) except for one open-ended question asking participants about experiences with a traumatic injury. The first question asked respondents whether they have completed resuscitation training and at what level. The next two questions polled personal experiences with traumatic injury. Six questions were then included to measure knowledge of Stop the Bleed® techniques and major concepts. Accuracy on these six questions was a key indicator for Stop the Bleed® proficiency.

There are two files included in the repository (Table 1). The first is the actual survey instrument used to collect the data [3]. The second file is the actual dataset. The dataset contains the following demographic information: age range, sex, race, US state of respondents’ address, marital status, number of children if any, education, employment status, and income level. The dataset also contains responses to every individual question on the survey instrument.

**Table 1:** Direct URL to data: 10.7303/syn51109429.1

Label	Name of data file/data set	File types (file extension)	Data repository and identifier (DOI or accession number)	Reference number
Data file 1	Data identification number for dataset:	MS Excel file (.xlsx)	Repository name: Synapse 10.7303/syn51109429.1	1
Data file 2	Data identification number for survey instrument:	MS Excel file (.xlsx)	Repository name: Synapse 10.7303/syn51109431	3

## Survey results

The study included 200 participants, of which 111 were female (55.5%) and 89 were male (44.5%). The age of respondents ranges from 16 to 80 years, with a median of 38 years and an interquartile range of 30–46 years. Participants are heterogenous by race: 132 were white or Caucasian (66%), 13 were Hispanic (6.5%), 22 were Black or African American (11%), 13 were Asian (6.5%), and 11 preferred not to say or other (5.5%). By employment type, 95 are wage-employed (47.5%), 22 are self-employed (11%), 12 are retired (6%), 13 are unemployed but seeking work, 11 are students (6.5%), 17 work within the home (8.5%), and 19 are unable to work or other (9.5%). Income status is categorized using the following tier system: high iii (7%), high ii (3.5%), high i (4.5%), middle ii (10%), middle i (11.5%), lower ii (32%), and lower i (25.5%). These tiers correspond to the following levels of income per year: high iii over $150,000, high ii between $125,000 and $149,999, high i between $100,000 and $124,999, middle ii between $75,000 and $99,000, middle i between $50,000 and $74,999, lower ii between $25,000 and $49,999, and lower i less than $24,999.

### Level of resuscitation training

**Fig. 1:**
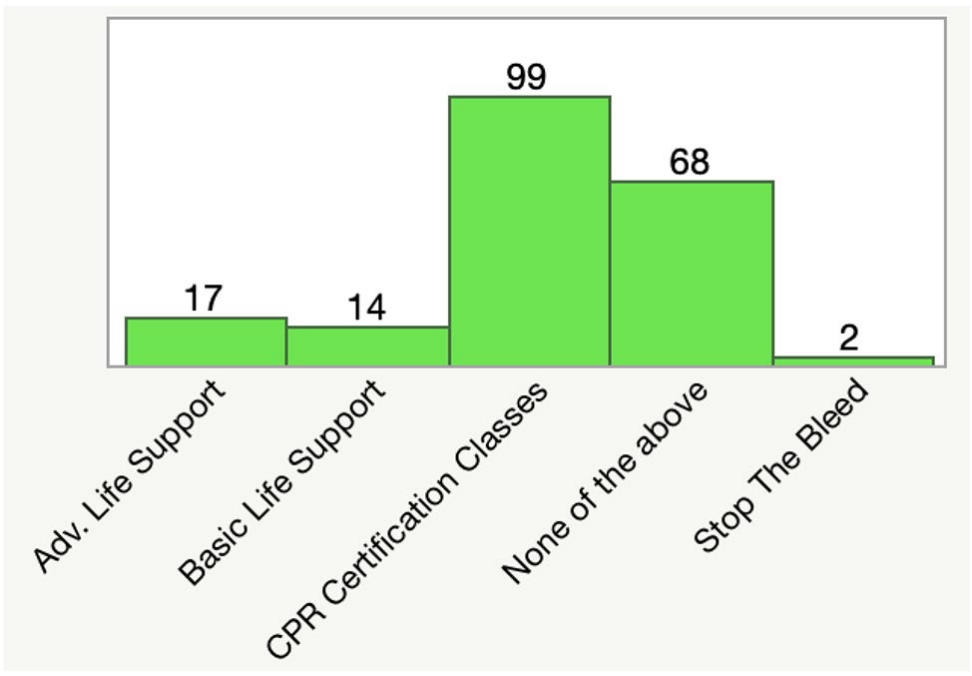
Participants’ certifications in resuscitation training

Participants were polled on their experience with completed resuscitation instruction in the first question of the survey. The most frequently selected response was completion of a CPR certification class (49.5%). This was followed by “none of the above” (34%), Advanced Life Support (8.5%), Basic Life Support (7%), and Stop the Bleed (1%). This distribution is visualized below (Fig. 1).

### Performance on stop the Bleed® Assessment

**Fig. 2:**
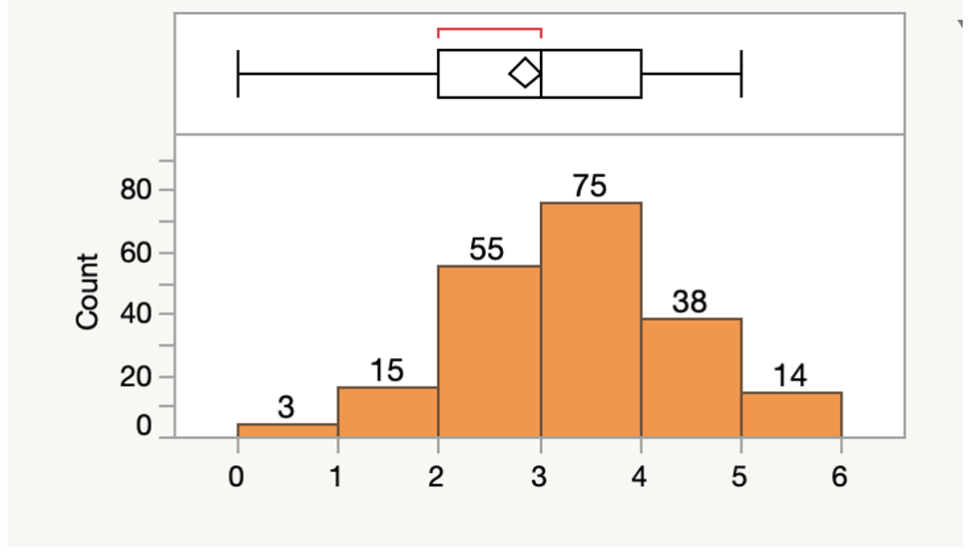
Distribution of correct responses on Stop the Bleed® quiz

Six questions were included in the questionnaire as a mini-assessment to evaluate participants’ working knowledge of Stop the Bleed® techniques. On average, respondents answered 2.860 ± 0.076 questions correct with a minimum of zero and maximum of five correct responses. The interquartile range was two to four correct answers. These findings are displayed below (Fig. 2).

**Fig. 3:**
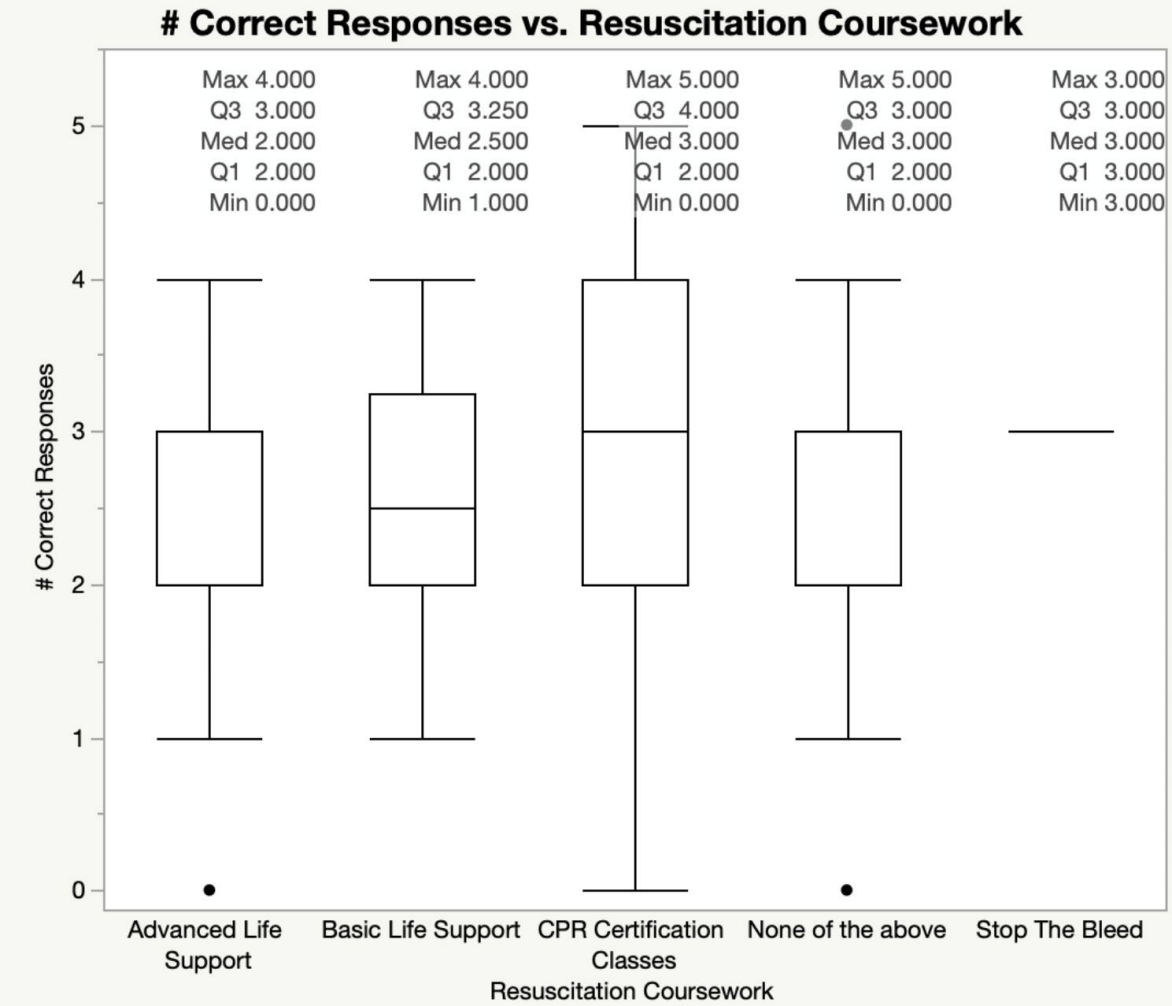
Box plots of Stop the Bleed® quiz performance by resuscitation training

The cohort’s performance on the Stop the Bleed® mini-quiz was further visualized according to participants’ level of resuscitation training (Fig. 3).

### Factors related to higher quiz performance

Participants’ demographic characteristics and resuscitation coursework history were included as variables in a multivariate regression to measure success on the quiz questions. While age (p = 0.498), gender (p = 0.982), race (p = 0.401), and education (p = 0.063) were not statistically significant, resuscitation coursework (p = 0.004) and income (p = 0.049) were important determinants of Stop the Bleed® knowledge. More specifically, participants with CPR certification (p = 0.020) and/or a postgraduate degree (p = 0.015) scored higher than their counterparts in this sample cohort.

## Discussion

Stop the bleed® (STB) training is important, as it places emphasis on circulation control in the potentially exsanguinating patient [4]. This critical concept is actually incorporated into the latest CPR guidelines. Participants in the current study who had prior CPR training performed better on the STB quiz, which makes sense as hemorrhage control complements CPR training.

While the current study was only performed with English language participants, STB has actually been translated into four languages including Arabic, Burmese, Somali, and Swahili, after culturally adapting the materials [5]. It is also available in Spanish and Ukranian, making that a total of 6 languages that STB training can be delivered in. From 2017 to 2022, the course has been taught in 6 different languages in 138 countries, resulting in more than 120,000 courses. As a result of these courses, more than 2 million people have been trained worldwide, which includes more than 105,000 instructors [6].

A systematic review of STB training includes 36 trials comprising 11,561 trainees found that correct uses of tourniquets significantly increased after the STB training. The willingness to apply a hemostatic dressing in a real-world situation also increased significantly [7].

STB represents a simple, life-saving treatment that can be delivered in many formats- a study of 106 participants demonstrated that it can even be delivered fully remotely [8]. While versatile and accessible, a 2022 systematic narrative review identified several ways the course can be further improved. These included providing teaching technique materials for instructors, having a refresher course, broadening the list of eligible instructors, and maintaining some aspect of instructor-led training, amongst others [9].

### Limitations

The limitations of these data are these inherent to any dataset based on survey responses. In particular, respondents may have tried to look up answers in order to be able to answer some of the questions. These data could be used to plan training courses and especially assessment tools for this import hemorrhage control and first aid training.

## Data Availability

The data described in this Data note can be freely and openly accessed on [Synapse] under [DOI 10.7303/syn38269688.1] after registration as a synapse user at http://synpase.org. Please see Table 1 and references [1, 3] for details links to the data.
